# Physical Exercise Intervention Characteristics and Outcomes in Frail and Pre-Frail Older Adults

**DOI:** 10.3390/geriatrics9060163

**Published:** 2024-12-16

**Authors:** María Caicedo-Pareja, Diego Espinosa, Jennifer Jaramillo-Losada, Leidy T. Ordoñez-Mora

**Affiliations:** Health Faculty, Physiotherapy Program Universidad Santiago de Cali, Cali 760035, Colombia; maria.caicedo15@usc.edu.co (M.C.-P.); diego.espinoza00@usc.edu.co (D.E.); jennifer.jaramillo01@usc.edu.co (J.J.-L.)

**Keywords:** frailty, physical exercise, physiotherapy, geriatrics, aged

## Abstract

(1) Background: Frailty is a multifactorial syndrome that significantly impacts the functional abilities of older adults, making them more vulnerable to falls, disabilities, and dependence. Exercise can serve as an effective intervention for pre-frail and frail older adults, improving muscle strength and reducing the risk of falls. This research aims to clarify the physical exercise protocols and their outcomes for this population. (2) Methods: A scoping review was conducted to summarize the evidence on physical activity parameters for frail and pre-frail older adults. The search included primary evidence sources published in PubMed, PEDro, Biomed, Scopus, and Springer, as well as search engines like Google Scholar and Dialnet. The keywords used were ([frailty] OR [frail] AND [exercise]). The PEDro and MINORS scales were used to assess the quality of the evidence and evaluate the risk of bias. (3) Results: Eighteen studies met the eligibility criteria. The most commonly reported exercise program was multicomponent, which included aerobic activities at 70% of the maximum effort and strength exercises at 20% to 80% of the participants’ maximum capacity. This approach proved effective for this population. (4) Conclusions: The studies suggest that exercise is a successful intervention strategy for addressing frailty. However, not all the articles provided adequate information regarding the dosing of their interventions.

## 1. Introduction

Frailty is a multifactorial geriatric syndrome characterized by a complex pathophysiological process. It involves a reduction in physiological reserves, an increased risk of functional decline, and greater vulnerability to adverse events, such as falls [1], disability [2], delirium [3], hospitalization, institutionalization, and, in severe cases, death. As a result, frailty can lead to unfavorable episodes and heightened dependency in performing activities of daily living [4,5].

It is estimated that the global population aged 65 and over will rise from 10% in 2022 to 16% by 2050. This demographic shift can be attributed to increased life expectancy, with some individuals reaching 90 years or even becoming centenarians, as well as advances in the detection and treatment of chronic diseases [6]. Frailty affects a significant proportion of older adults. As people age, their mobility often declines due to various pathophysiological factors, hospitalization, or the natural aging process [7], which can lead to systemic complications. A study conducted in 2019 estimated the prevalence of frailty among older adults to be 17.9%, with rates ranging from 7.7% to 42.6% reported in Latin America and the Caribbean [8]. This highlights the critical need to understand this syndrome better and implement targeted interventions within this population.

Models have been developed to assess frailty and pre-frailty [9,10,11,12,13,14], incorporating criteria such as involuntary weight loss, weakness, fatigue, reduced endurance, slow movement, and low levels of physical activity [2]. Identifying these criteria is crucial for targeting interventions to address or reverse the physical aspects of frailty. Doing so can help reduce long-term adverse outcomes and preserve quality of life [15].

Various interventions have been employed to address frailty, including nutritional strategies, which have shown mixed results due to intrinsic and extrinsic factors [16,17,18], and multicomponent physical exercise and personalized healthcare, which have demonstrated benefits [19,20,21,22]. Exercise has been associated with lower risks of mortality, institutionalization, and functional decline [23,24,25]. Three systematic reviews further stress that exercise improves mobility, strength, and physical function in frail and pre-frail adults. Treacy et al. (2022) [26] report that the mobility benefits last up to six months, while Lim et al. (2024) [27] emphasize its role in increasing the likelihood of reversing pre-frailty. Eidam et al. (2024) [28] confirm that exercise reduces frailty, although heterogeneity and bias in studies limit the generalizability of these findings. This highlights the need for standardized, replicable exercise-based interventions with clear parameters, dosage, and progression to ensure consistency across care settings.

To enhance understanding in this field, this review aims to identify the parameters and characteristics of physical exercise and the outcomes achieved in frail and pre-frail older adults, providing an integrated perspective on its effects.

## 2. Materials and Methods

We conducted a scoping review to provide a comprehensive overview of the existing evidence on the parameters of physical exercise in frail and pre-frail individuals, particularly focusing on aspects such as strength, cardiovascular endurance, flexibility, and balance.

To this end, we employed the criteria outlined in the PRISMA Scoping Review strategy [29] and followed the methodology prescribed by the Joanna Briggs Institute [30] and the reported guides [31]. This involved the use of databases and search engines for evidence collection.

### 2.1. Sources of Information

The search covered databases such as PubMed, PEDro, Biomed, Scopus, and Springer, as well as search engines like Google Scholar and Dialnet. Only the sources of evidence published in English, Spanish, and Portuguese were considered for inclusion. The search was conducted until 20 July 2023 using the search terms ([fragility] OR [frail] AND [exercise]) across all the databases, and related articles were identified through a manual search.

### 2.2. Selection of Sources of Evidence

The process of article selection was structured into four phases:

Step 1: A screening was conducted through systematic searches.
Step 2: The entire team reviewed the sources using the eligibility criteria and the definition/elaboration document.
Step 3: The team met to discuss discrepancies and change the eligibility criteria and the definition/elaboration document.
Step 4: The team only began evaluation when a 100% agreement was reached.

Screening adhered to the inclusion criteria outlined in the review protocol. The selection of sources, both in terms of title/abstract and full-text selection, was conducted independently by three reviewers. Any disagreements were resolved through consensus or, if necessary, by the decision of a fourth reviewer.

The research question was framed as follows:

- Population: adults with frailty or pre-frailty over 65 years old.
- Intervention: any physical exercise modality.
- Comparison: conventional management and no intervention or other intervention modality.
- Outcomes: measures related to frailty, physical capacity, and independence/autonomy.

The following question, therefore, arises: what are the protocols based on any type of exercise (without the addition of any other intervention, supplementation, or nutritional support), and what are their results in frail and pre-frail elderly people?

#### 2.2.1. Inclusion Criteria

- Papers published in English, Spanish, and Portuguese.
- Studies with observational research methodology and clinical trials.

#### 2.2.2. Exclusion Criteria

- Presence of cognitive impairment.
- Papers with low methodological quality measured using the MINORS scale [32] and the Pedro scale [33].
- Gray literature, such as doctoral theses, undergraduate and graduate theses, and scientific reports.
- Inclusion of other dietary guidelines, recommendations, or nutritional supplementation in addition to exercise.

Data extraction was performed using an Excel template to capture pertinent information from each study. This template included details about the study and the parameters of the physical exercise intervention, which were intervention period, intensity, density, progression, frequency, and primary results as per the assessed measures.

#### 2.2.3. Quality Appraisal

The evaluation of quality and risk of bias was carried out using the PEDro scale for clinical trials [33], which considers elements such as randomization, allocation concealment, blinding, initial comparability, and intention-to-treat analysis. For non-randomized studies, the MINORS scale (Methodological Index for Non-Randomized Studies) [32] was used. This scale is based on 12 items for comparative studies or 8 items for non-comparative designs, including criteria such as the study objective, inclusion of consecutive patients, adequate follow-up, balanced endpoints, and unbiased evaluation.

## 3. Results

### 3.1. Description of Evidence Characterization

**Figure 1:**
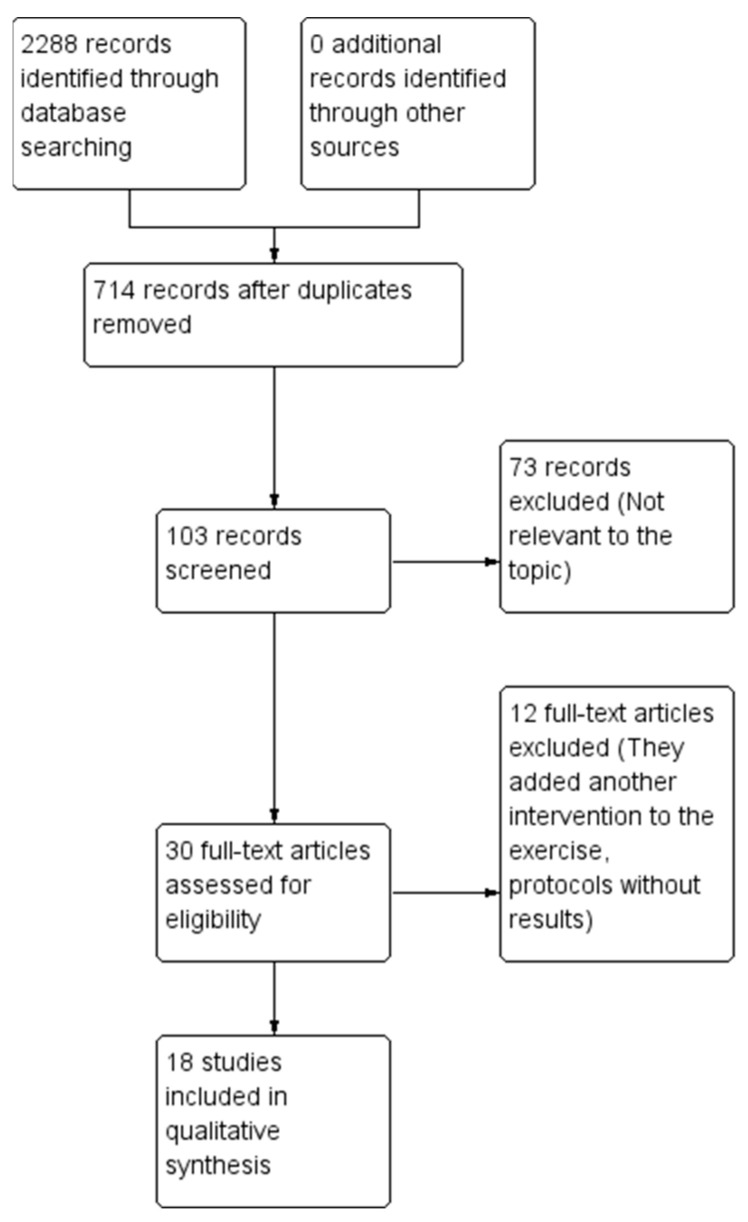
Flow chart for the scope review process adapted from the PRISMA statement Source: Modified from Moher et al., (2009) [34].

A total of eighteen papers were included in the review (see Table 1) utilizing various research methodologies such as clinical trials and both prospective and retrospective intervention studies (refer to Figure 1). The study participants consisted of individuals aged 65 years and older, with group sizes ranging from 20 to 100 participants. The types of exercise implemented in the interventions included bodyweight training, machine training, lower-extremity resistance exercises, and multicomponent exercise programs that combined aerobic endurance, muscular strength, balance, and flexibility. Additionally, there were multisystem exercise programs, which encompassed proprioception training, muscular strength training, reaction time training with auditory signals, and postural balance training. Other forms of exercise included power training, high-intensity interval training, elastic band exercises, strength training, combined exercise and nutrition interventions, and cognitive exercises.

**Table 1:** Summary characteristics of included studies.

Author	Year	Country	Study Type	Condition	IG Population	CG Population	IG Intervention	CG Intervention
Lai et al. (2021) [35]	2021	China	RCT	Hospitalized older adults with pre-frailty	N = 30	N = 30	Lower-extremity resistance exercise	Routine care
Chittrakul et al. (2020) [36]	2020	Thailand	RCT	Pre-frail older adults in the community	N = 36	N = 36	Multisystemic physical exercise program	Flexibility exercise training Participants met with a researcher at the primary care unit once a week during the study to share their health experiences.
Sadjapong et al. (2020) [37]	2020	Thailand	RCT	Frail older adults in the community	N = 32	N = 32	Multicomponent exercise	Regular care provided by the general practitioner and specialist doctor who would be available to the participants.
Losa-Reyna et al. (2019) [38]	2019	Spain	Controlled, nonrandomized, quasi-experimental intervention study	Frail older adults in the community	N = 11	N = 9	Power training + HIIT	Participants were advised not to change their eating habits or physical activity during the course of the study.
Chen et al. (2019) [39]	2019	China	RCT	Pre-frail older adults in the community	N = 35	N = 35	Exercise program with elastic bands	Participants maintained normal daily activity and did not receive any special intervention.
Ng et al. (2015) [40]	2015	Singapore	RCT	Community-living pre-frail and frail old adults	N = 48	N = 50	Three interventions: physical, nutritional, and cognitive training	Access to standard health and older adults care services, including primary and secondary level care from government or private clinics and hospitals, community-based social rehabilitation, and recreational and daycare services.
Cardalda et al. (2019) [41]	2019	Spain	RCT	Frail institutionalized older adults	TG: 25 MG: 23 N = 48	N = 29	The TG followed a strength program with TheraBands. The MG followed a multicalisthenics exercise program.	Participants did not perform any physical exercise; they performed activities required by the institutional center, such as handicrafts, reading comprehension, and cognitive stimulation.
Costa et al. (2020) [42]	2020	Brazil	RCT	Frail older women living in the community	CB N = 14	H + CB N = 11	Multicomponent physical training program (CB)	Multicomponent physical training program (H + CB)
Sahin et al. (2018) [43]	2018	Turkey	Prospective, randomized, controlled design	Institutionalized frail elderly	Hi N = 16 Li N = 16	N = 16	High- and low-intensity strength training	Participants did not perform any type of exercise but were told to continue with their usual daily routine.
Concha-Cisternas et al. (2020) [44]	2020	Cuba	Pre-experimental, longitudinal study	Institutionalized frail elderly	N = 28	NR	Multicomponent physical training program	NR
Buendía-Romero et al. (2020) [45]	2020	Spain	Pre-experimental study	Pre-frail and frail	N = 14	NR	Multicomponent physical training program	NR
Kao et al. (2022) [46]	2022	Taiwan	Clinical trial	Pre-frail community-dwelling older adults	N = 15	N = 16	Vibration exercise plus multicomponent program	Multicomponent exercise program
Bray, N. et al. (2020) [47]	2020	Canada	Quasi-Experimental, Pilot Study	Pre-frail frailty community	N = 9	N = 12	Multicomponent physical training program	The group maintained their normal routine for the same duration
Chan and Yu (2022) [48]	2022	Hong Kong	Randomized controlled trial	Frailty community	N = 61	N= 63	Moderate-intensity low-impact stepping exercise program	Health-education program
Dun et al. (2022) [49]	2022	China	Randomized controlled trial	Pre-frail community	N = 22	N = 21	X-CircuiT circuit training	will be given one-time advice on physical activity according to current guidelines
Lustosa et al. (2011) [50]	2011	Brazil	Randomized crossover trial	Pre-frail community	N = 32	N = 16	Resistance exercise program	Participants were instructed to remain with the same activities of normal life, without performing any training Started training the same way as the first group after ten weeks.
Meng et al. (2020) [51]	2020	China	Randomized controlled trial	Pre-frail frailty community	N = 74	N = 72	Supervised exercise training	The participants in HEG attended a 15 min session of home-based exercise instructions.
Coelho-Júnior and Uchida (2021) [52]	2021	Brazil	Randomized parallel controlled tri	Pre-frail frailty community	LSRT: 26 HSRT: 26	N = 26	Low-speed resistance training and high-speed resistance training	Flexibility sessions

N: number; IG: intervention group; CG: control group; NR: no response; TG: TheraBand group; MG: multicalisthenic group; H: home; CB: center; RCT: randomized controlled trial; HRQOL: fall prevention and health-related quality of life; MPE: multisystem physical exercise; Hi: high intensity; Li: low intensity; HIIT: high-intensity interval training. NC: not classified.

### 3.2. Risk of Bias Assessment

To evaluate the risk of bias in the studies included in this review, we utilized the MINORS scale [32] (see Table 2a). This scale features 12 specific items aimed at assessing the validity of observational studies. Additionally, we applied the PEDro scale [33] (see Table 2b), which consists of 10 items specifically designed for clinical trials. The assessment based on these scales indicated that the papers exhibited a commendable level of methodological quality according to the criteria established by these tools.

### 3.3. Types of Training and Physical Qualities Implemented in Physical Exercise for Frail and Pre-Frail Older Adults

Eight studies primarily focused on strength, power, and muscular endurance training [35,39,40,41,43,48,50,52]. Among these, two studies specifically highlighted the use of elastic bands (TheraBands) in their interventions, emphasizing strength development as a key component [39,41]. Additionally, nine studies adopted a multicomponent training approach that targeted various physical qualities, including aerobic endurance, muscle strength, balance, and flexibility [37,38,39,42,44,45,47,49,51]. One study examined proprioception, muscle strength, reaction time, and balance [36]; another focused on strength and lower extremity balance [40]; and one included multicomponent training with vibration [46]. The findings indicate that muscular strength training is one of the most frequently utilized methods, while multicomponent training is a widely recommended strategy for frail older adults. (Supplementary Material Table S1: Type of Training, Physical Exercise Parameters, and Physical Qualities Implemented)

### 3.4. Physical Exercise Parameters for Frail and Pre-Frail Older Adults

Regarding the intervention parameters for both the intervention and control groups (see Supplementary Material Table S1), only 5 out of the 18 included studies had a control group that performed physical exercise [36,42,46,51,52]. These studies were further categorized into two types: flexibility exercise training [52] and multicomponent training programs, which were conducted either in a facility [36,42,46] or at home [51]. Additionally, four studies included a secondary intervention that focused on social services accompaniment [40], cognitive stimulation [41], health education [48], or health counseling [39]. In contrast, six studies indicated that participants should continue with their usual activities while having access to standard medical care (both primary and secondary), but without any special interventions [35,37,38,43,47,50]. Two studies did not include a control group for comparison [44,45].

The intervention durations across the studies ranged from 3 to 24 weeks, with 12 weeks being the most common duration. All the studies reported significant short-term improvements for interventions lasting 3 to 8 weeks [38,39,43,44,45], as well as long-term improvements for those lasting 12 to 24 weeks [35,37,40,42,46,47,49]. The intensity of the interventions varied between studies, and not all provided specific intensity levels [36,39,41]. Among those that did report intensity, the Borg scale or maximum heart rate was used to measure intensity during aerobic exercises [42,44,47,50]. One study monitored intensity using an electrocardiogram [49], while strength training intensity was quantified based on one repetition maximum (1 RM), which was set at 20% to 80% of 1 RM [37,38,40,43,44,47,50,51,52]. Regarding density and progression, not all the studies reported progression details [30,35,36,40,42,43,46,48]. Those that did provide progression data specified month-by-month or week-by-week details, often involving increases in intensity [37,38,41,44,51,52]. Density was individualized in each study, with specifications for contraction time or posture maintenance; however, one study did not report density values [40]. The intervention sessions ranged from 30 to 90 min in duration, with frequencies of 1 to 5 times per week, and 3 times per week being the most common. Finally, the number of sets per exercise ranged from 1 to 4, with repetitions varying from 6 to 30.

### 3.5. Tests and Measures Used for Frailty Reversal

Table 3 indicates that eight studies utilized Fried’s criteria to assess frailty levels in older adults. These studies supplemented Fried’s criteria with additional tests and measures, which revealed a significant reversal in the frailty levels [37,38,44,48,49]. However, studies [40,42,47], while also using this measure, did not provide enough information to determine their effectiveness.

**Table 3:** Reported test and measures; description of outcome measures before and after the intervention.

Author	Tests and Measurements	Result Before Intervention			Result After Intervention		Significance
Lai et al. (2021) [35]	6 min walk test	IG: 548.04 SD: 74			653.18 SD: 59.79		*p*: >0.001 *
		CG: 559.68 SD: 67.05			548.8 SD: 63.87		*p*: <0.001 *
	30 s standing test	IG: 12.87 SD: 3.31			17.21 SD: 3.95		*p*: 0.034 *
		CG: 13.16 SD: 4.1			13.43 SD: 4.22		*p*: <0.001 *
	8 feet up and go	IG: 5.80 SD: 0.83			4.71 SD: 0.8		*p*: 0.374 *
		CG: 5.51 SD: 0.49			5.29 SD: 0.67		*p*: <0.001 *
	Barthel index	IG: 80.3 SD: 10.6;			NR		*p*: 0.094
		CG: NR.			NR		
	Muscle strength	IG: 5.76 SD: 0.8			8.04 SD: 0.96		*p*: <0.001 *
		CG: 6.11 SD: 1.15			6.04 SD: 0.92		*p*: <0.001 *
Chittrakul et al. (2020) [36]	Fall risk index score	IG: 1.99 SD: 0.58			1.13 SD: 0.84		*p*: <0.001 *
		CG: 1.97 SD: 0.61			2.22 SD: 0.55		*p*: <0.05 *
Sadjapong et al. (2020) [37]	Grip strength	IG: 15.71 SD: 6.21			18.84 SD: 5.01		*p*: 0.08
		CG: 15.50 SD: 6.47			16.70 SD: 8.05		*p*: 0.08
	Berg Balance Scale (BBS)	IG: 49.12 SD: 3.58			52.68 SD: 3.49		*p*: <0.01 *
		CG: 49.96 SD: 4.40			44.46 SD: 9.52		*p*: <0.01 *
	Timed up and go (TUG)	IG: 12.21 SD: 2.26			10.33 SD: 2.91		*p*: <0.01 *
		CG: 12.43 SD: 5.04			15.75 SD: 6.96		*p*: <0.01 *
	Frailty score	IG: 3.18 SD: 0.39			1.65 SD: 0.86		*p*: <0.01 *
		CG: 3.25 SD: 0.50			3.09 SD: 0.92		*p*: <0.01 *
Losa-Reyna et al. (2019) [38]	Fried criteria	IG: 3.1 SD: 1.1			1.5 SD: 0.8		*p*: <0.001 *
		CG: 2.3 SD: 1.4			2.6 SD: 1.3		*p*: <0.001 *
	10 m walk test	0.56 SD: 0.17			0.72 SD: 0.12		*p*: 0.001 *
		0.64 SD: 0.16			14.8 SD: 4.0		*p*: <0.001 *
	SPPB	6.8 SD: 1.5			9.8 SD: 1.5		*p*: <0.001 *
		7.4 SD: 2.0			6.9 SD: 2.7		*p*: <0.001 *
	Usual walking speed	IG: 2.1 SD: 1.0			2.8 SD: 0.8		*p*: <0.03 *
		CG:2.5 SD: 0.9			2.3 SD: 1.0		*p*: 0.03 *
	Chair support test	IG:15.6 SD: 2.7			10.8 SD: 2.5		*p*: <0.001 *
		CG:15.7 SD: 3.0			14.8 SD: 4.0		*p*: <0.001 *
	6 min walk test	IG: 257.4 SD: 61.7			302.1; SD: 71.8		
		CG: NR					
Chen et al. (2019) [39]	Fried criteria	NR					
	Grip strength	IG women: 16.73 SD: 2.42 Men: 25.90 SD: 3.06			21.76 SD: 2.84 30.76 SD: 4.11		*p*: 0.000 *
		CG women: 16.08: SD: 2.84 CG Men: 26.77 SD: 2.44			15.73 SD: 2.61 26.79 SD: 2.26		*p*: 0.010 *
	Walking speed	IG: 5.59 SD: 0.91			4.32 SD: 0.57		*p*: 0.000 *
		CG: 5.98 SD: 0.95			5.98 SD: 0.97		*p*: 0.000 *
	Physical activity	IG: 20 (60, 6)			31 (93, 9)		
		CG: 19 SD: 57.6			22 SD: 66.7		
Ng et al. (2015) [40]	Frailty score	IG: 2.2 SD: 0.85			1.4 SD: 0.80		
		CG: 1.8 SD: 0.80			1.6 SD: 0.97		
	Knee extension strength	IG: 14.1 SD: 4.63			15.5 SD: 5.19		
		CG:15.5 SD: 4.73			14.8 SD: 4.47		
	Walking speed	GI: 6.1 SD: 2.08			4.9 SD: 0.99		
		CG: 176.9 SD: 111.0			209.7 SD: 123.3.		
Cardalda et al. (2019) [41]	Barthel index	GI TG: 69.60; SD: 26.10 MG: 56.09 SD: 24.45			TG: 72.00 SD: 25.45 MG: 58.68 SD: 24.46		
		GC: 58.45 SD: 29.55			55.80 SD: 24.69		
	FTSTS	GI: 16.44; SD: 4.52 MG: 16.63; SD: 4.07 CG: 15.34; SD: 3.34			13.96 SD: 5.23 14.26 SD:4.66 16.39 SD: 7.06		
		CG: 15.34 SD: 3.34			16.39 SD: 7.06.		
Costa et al. (2020) [42]	Fried’s criteria score	IG: 11 CG:NR			0		
Sahin et al. (2018) [43]	SPPB	LI: 3.5 (1–5)			LI: 7 (1–9)		*p*: 0.001 *
		HI: 4 (1–6)			HI: 8(3–11)		<0.0001 *
	Barthel index Barthel index	CG: 4.5 (1–7)			CG: 4 (1–7)		*p*: 0.038 *
		LI: 80 (70–85)			85 (65–90)		*p*: <0.001 *
	Lawton and Brody	HI: 85 (65–90)			HI: 92.5 (75–100)		*p*: <0.001 *
		CG: 85 (75–95)			CG: 85 (70–95)		*p*: 0.157
	Knee extension strength Handgrip strength	CG: 11.5 (7–14)			CG: 11 (7–14)		*p*: 0.059 *
		LI: 5.66			LI: 9.22		*p*: <0.001 *
	SPPB	LI: 3.5 (1–5) HI: 4 (1–6)			LI: 7 (1–9) HI: 8(3–11)		*p*: 0.001 * <0.0001 *
Concha-Cisternas (2020) [44]	Fried criteria	IG: 2.87 SD: 1.12 CG:NR			2.00 SD: 0.92		*p*: 0.007 *
Buendía-Romero et al. (2020) [45]	SPPB	IG: 4.3 SD: 3.5 CG: NR			7.1 SD: 3.9		*p*: 0.001 *
	Manual pressure	Dominant hand: 21.09 SD: 9.2 Non-dominant hand: 19.7 SD: 8.5 CG:NR			22.08; SD: 8.9 20.06; DS: 7.2		*p*: 0.2 *p*: 0.2
	Barthel disability	IG: 76.3 SD: 21.4			82.1; D: 17.5		*p*: 0.1
	Lawton disability	IG: 3.3 SD: 2.2			5.4 DS: 1.8		*p*: 0.013 *
Kao et al. (2022) [46]	Daily life activities	IG: 100 SD: 0			100 SD: 0		*p*: 0.001 *
		CG: 98.8 SD: 2.9			97.5 SD: 8.8		*p*: <0.001 *
	Grip strength	IG: 18.9 SD: 4.9			20.2 SD: 4.1		*p*: 0.001 *
		CG: 15.3 SD: 5.1			18.4 SD: 4.1		*p*: 0.54
Bray, N. et al. (2020) [47]	Frailty phenotype (FP)	IG: (1, 14) = 8.5 CG: NR		NR			
	Clinical frailty scale (CFS)	IG: (1, 14) = 4.8 CG: NR			NR		
	Gait speed (GS)	NR			IG: (1, 7) = 15.2, *p* ≤ 0.01 CG: NR		
	Handgrip strength	NR			IG: (1, 6) = 17.3, *p* ≤ 0.01 CG: NR		
	Sit to stand (STS)	IG: (1, 13) = 4.8, *p* ≤ 0.05 CG: (1, 13) = 6.6, *p* = 0.02			IG: (1, 6) = 18.2, *p* ≤ 0.01 CG: (1, 13) = 0.7, *p* = 0.4		
	Muscle strength performance	IG: (1, 7) = 5.9, *p* ≤ 0.05 CG: NR			NR		
	Muscle strength performance isotonic velocity	IG:(1, 7) = 17.5, *p* = ≤ 0.01 CG: NR *			IG: (1, 13) = 14.8, *p* ≤ 0.01 CG: (1, 7) = 21.7, *p* ≤ 0.01		
Chan And Yu (2022) [48]	MFI-20	IG: T0 62.9 ± 9.4			IG: T1 52.4 ± 12.9 T2 55.1 ± 12.2		*p*: <0.001
		CG: T0 63.1 ± 9.6			CG: T1 63.9 ± 9.4 T2 64.5 ± 10.2		
	Frail scale	IG: T0 1.93 ± 1.01			IG: T1 1.00 ± 1.06 T2 1.10 ± 1.13		*p*: <0.001
		CG: T0 2.10 ± 1.12			CG: T1 2.12 ± 1.20 T2 2.19 ± 1.15		
	IPAQ	IG: T0 4995.6 ± 2804.2			IG: T1 6136.0 ± 2838.4 T2 6788.3 ± 3099.7		*p*: 0.27
		CG: T0 4516.4 ± 2208.4			CG: T1 4798.7 ± 2492.7 T2 5109.8 ± 2486.6		
	Two- minute walk	IG: T0 83.1 ± 23.9			IG: T1 92.8 ± 25.7 T2 87.2 ± 22.6		*p*: 0.035
		CG: T0 81.7 ± 24.8			CG: T1 83.1 ± 24.3 T2 75.3 ± 25.2		
	Profile of Mood State (POMS)	IG: T0 18.5 ± 10.9			IG T1 12.8 ± 8.3 T2 14.0 ± 8.7		*p*: 0.0033
		CG: T0 18.3 ± 11.0			CG: T1 15.6 ± 8.2 T2 17.2 ± 9.7		
Dun et al. (2022) [49]	Fried frailty score- Senior fitness	Pre mean ± SD			Mean change [95%CI]		*p*: <0.001
		IG: 1.9 ± 0.3			IG: −1.7 [−2.0 to −1.5]		
		CG: 1.9 ± 0.4			CG: 0 [−0.2 to 0.2]		
	6 min walk distance, meters	IG: 519 ± 61			IG: 68 [50 to 86]		*p*: <0.001
		CG: 515 ± 103			CG: −16 [−50 to 18]		
	Muscular strength	30 s arm curl test, reps			30 s arm curl test, reps		*p*: <0.001
		IG: 20 ± 3 CG: 21 ± 5			IG: 8 [7 to 9] CG: −1 [−3 to 1]		
		30 s chair stand test, reps			30 s chair stand test, reps		*p*: <0.001
		IG: 19 ± 4 CG: 18 ± 4			IG: 5 [3 to 7] CG: −1 [−3 to 0]		
	Flexibility	Back scratch test, cm			Back scratch test, cm		*p*: <0.001
		IG: −4 ± 9 CG: −1 ± 9			IG: 7 [4 to 10] CG: −4 [−7 to −1]		
		Chair-sit-and-reach test, cm			Chair-sit-and-reach test, cm		
		IG: 4 ± 11 CG: 1 ± 10			IG: 8 [5 to 11] CG: −2 [−7 to 2]		
	Agility and dynamic balance	2.4 m up-and-go, seconds			2.4 m up-and-go, seconds		*p*: <0.001
		IG: 6.7 ± 0.9 CG: 6.6 ± 1.1			IG: −1.1 [−1.4 to −0.8] CG: 0.7 [0.1 to 1.3]		
		Single leg stance test, seconds			Single leg stance test, seconds		
		IG: 19.4 ± 19.7 CG: 22.5 ± 18.9			IG: 20.7 [11.4 to 30.1] CG: −1.3 [−8.2 to 5.7]		
Lustosa et al. (2011) [50]	Timed up and go	IG: 11.09 (2.3) CG: 10.81 (2.4)			IG: 10.41 (1.9) CG: 10.09 (1.7)		*p*: 0.01 *
	Gait speed	IG: 4.85 (0.7) CG: 4.90 (1.1)			IG: 4.36 (0.7) CG: 4.87 (0.8)		*p*: 0.01 *
	Work/weight at 60%	IG: 119.16 (36.6) CG: 122.49 (43.1)			IG: 122.36 (33.2) CG: 128.95 (38.8)		*p*: 0.07
	Work/weight at 180 (%)	IG: 77.79 (26.8) CG: 76.28 (26.2)			IG: 83.14 (24.0) CG: 84.48 (28.3)		*p*: 0.02 *
	Power at 60%	IG: 44.78 (12.7) CG: 40.16 (12.5)			IG: 45.55 (10.7) CG: 46.00 (11.2)		*p*: 0.06
	Power at 180%	IG: 67.17 (20.4) CG: 58.38 (17.9)			IG: 72.66 (18.1) CG: 66.69 (18.2)		*p*: 0.02 *
Meng et al. (2020) [51]	Walking speed:	IG: 0.73 SD: 0.23 CG: 0.71 SD: 0.24			IG: 0.07 SD: 0.19 CG: 0.007 SD: 0.27		*p*: 0.009 *p*: 0.007
	Timed up and go test:	IG: 9.65 SD: 5.28 CG: 10.22 SD: 7.20			IG: −0.96 SD: 2.86 CG: 0.05 SD: 2.79		*p*: 0.008 *p*: 0.975
	Six-minute walking test	IG: 398.51 SD: 116.17 CG: 392.90 SD: 116.81			IG: 11.29 SD: 71.30 CG: 1.09 SD: 72.71		*p*: 0.210 *p*:0.975
	Single leg stance	IG: 3.89 SD: 3.47 CG: 4.02 SD: 3.19			IG: −0.03 SD: 5.45 CG: 0.34 SD: 2.77		*p*: 0.949 *p*:0.529
	Timed chair stance	IG: 6.38 SD: 3.01 CG: 6.19 SD: 4.05			IG: −0.53 SD: 1.88 CG: 0.01 SD: 1.47		*p*: 0.008 *p*: 0.994
Coelho-Júnior and Uchida (2021) [52]		Before intervention Pre-frail			Frail		
		LSRT	HSRT	CG	LSRT	HSRT	CG
	Frailty phenotype %	45.4	72.7	0	87.5	72.7	77.7
	Slow walking speed	18.1	45.4	20.0	87.5	81.8	66.6
	Unintentional weight loss	0	9.0	40.0	50	63.6	77.7
	Exhaustion	45.4	72.7	81.8	100	100	100
	Low activity level	0	9.0	20.0	100	100	100
	Right knee extensor kg	17.3 ± 4.2	11.7 ± 2.3	10.1 ±1.9	7.0 ± 1.9	7.1 ± 2.8	7.0 ± 5.7
	Left knee extensor kg	14.8 ± 3.1	12.3 ± 3.4	10.3 ± 2.3	6.6 ± 2.0	6.1 ± 3.7	6.6 ± 5.0
	Right hip flexor kg	11.1 ± 3.2	8.2 ± 3.3	8.6 ± 3.6	6.0 ± 1.7	5.4 ± 2.2	4.7 ± 2.8
	Left hip flexor kg	10.1 ± 2.7	8.1 ± 2.8	8.3 ± 2.5	5.4 ± 1.1	5.1 ± 2.5	4.3 ± 2.5
	Right ankle extensor	6.8 ± 2.1	6.4 ± 1.8	5.8 ± 1.1	5.6 ± 1.5	4.3 ± 2.6	3.8 ± 2.3
	Left ankle extensor	7.1 ± 1.7	6.4 ± 1.8	6.4 ± 1.1	3.8 ± 2.8	4.4 ± 2.4	3.7 ± 2.6
	Right one leg stand 30 seg	19.4 ± 9.7	10.9 ± 11.6	12.5 ± 12.0	0.1 ± 0.3	0.1 ± 0.4	2.2 ± 3.1
	Left one leg stand 30 seg	16.4 ± 11.0	13.0 ± 12.2	7.3 ± 10.4	0.0 ± 0.2	0.2 ± 0.4	2.3 ± 4.4
	Normal balance	10.0 ± 0.0	9.8 ± 0.6	10.0 ± 0.0	1.2 ± 3.5	1.8 ± 4.0	4.4 ± 5.2
	Semi tandem balance	10.0 ± 0.0	9.8 ± 0.6	10.0 ± 0.0	0.0 ± 0.0	1.0 ± 3.0	4.4 ± 5.2
	Tandem balance	10.0 ± 0.0	6.9 ± 0.6	10.0 ± 0.0	0.0 ± 0.0	0.8 ± 2.7	5.5 ± 5.2
	Sit to stand	8.4 ± 1.1	10.0 ± 2.3	8.0 ± 0.6	26.7 ± 11.6	26.2 ± 13.3	28.6 10.9
	Sit to stand concentric contraction	1.3 ± 0.2	0.8 ± 0.3	1.2 ± 0.2	0.35 ± 0.30	0.26 ± 0.20	0.17 ± 0.10
	Sit to stand eccentric contraction	1.1 ± 0.5	1.2 ± 0.4	1.3 ± 0.4	0.53 ± 0.51	0.53 ± 0.46	0.82 ± 0.46
	Timed up and go	8.0 ± 0.8	10.2 ± 2.7	6.2 ± 1.4	119.8 ± 180.2	20.8 ± 27.3	46.4 ± 36.3
	6 MWT	480 ± 137	460 ± 151	589 ± 179	150 ± 174	100 ± 136	91.4 ± 107
		After intervention					
		LSRT	HSRT	CG	LSRT	HSRT	CG
	Frailty phenotype	NR	NR	NR	NR	NR	NR
	Right knee extensor kg	19.2 ± 5.0	13.5 ± 3.5	9.8 ± 1.8	10.6 ± 5.3	7.9 ± 7.2	6.8 ± 5.2
	Left knee extensor kg	16.8 ± 4.3	14.3 ± 3.8	9.7 ± 2.3	9.7 ± 4.9	9.2 ± 7.5	6.2 ± 5.3
	Right hip flexor kg	12.8 ± 3.6	9.1 ± 3.7	8.1± 3.3	7.4 ± 2.9	6.6 ± 5.6	5.0 ± 3.0
	Left hip flexor kg	12.5 ± 3.9	8.7 ± 2.5	8.2 ± 2.6	6.8 ± 2.5	7.1 ± 3.8	4.7 ± 2.8
	Right ankle extensor	8.7 ± 2.9	7.6 ± 1.5	5.6 ± 1.3	6.1 ± 1.7	4.3 ± 4.0	3.8 ± 2.6
	Left ankle extensor	8.7 ± 2.7	7.5 ± 1.3	6.2 ± 1.3	4.5 ± 3.2	5.9 ± 3.0	3.2 ± 2.3
	Right one leg stand 30 seg	6.6 ± 0.7	14.2 ± 12.0	11.9 ± 12.2	1.0 ± 1.8	2.0 ± 5.7	2.8 ± 4.9
	Left one leg stand 30 seg	5.5 ± 0.9	17.9 ± 12.7	9.9 ± 11.5	0.2 ± 0.4	1.7 ± 5.0	3.3 ± 7.8
	Normal balance	10.0 ± 0.0	10.0 ± 0.0	10.0 ± 0.0	2.5 ± 4.6	2.7 ± 4.6	4.4 ± 5.2
	Semi tandem balance	10.0 ± 0.0	10.0 ± 0.0	10.0 ± 0.0	1.2 ± 3.5	1.8 ± 4.0	4.4 ± 5.2
	Tandem balance	10.0 ± 0.0	7.3 ± 4.2	10.0 ± 0.0	1.2 ± 3.5	0.9 ± 3.0	1.1 ± 3.3
	Sit to stand	6.6 ± 0.8	7.3 ± 2.0	1.1 ± 0.1	17.1 ± 11.7	18.9 ± 10.0	37.1 ± 19.3
	Sit to stand concentric contraction	1.7 ± 0.4	1.2 ± 0.4	1.2± 0.2	0.55 ± 0.50	0.50 ± 0.43	0.19 ± 0.0
	Sit to stand eccentric contraction	1.0 ± 0.4	1.0 ± 0.4	1.3 ± 0.4	0.65 ± 0.53	0.56 ± 0.46	0.86 ± 0.36
	Timed up and go	7.5 ± 0.8	9.4 ± 2.5	6.3 ± 1.4	64.2 ± 4.7.4	23.9 ± 20.1	48.7 ± 37
	6 MWT	589 ± 179	511 ± 135	478 ± 159	NR	NR	NR

Conventions: CG: control group; IG: intervention group; SD: standard deviation; CS: standing test; SPPB: Short Physical Performance Battery; FTSTS: five times sit-to-stand test; NR: not reported; F: female; M: male. Values are mean ± SD MFI-20: Multidimensional Fatigue Inventory; IPAQ: International physical activity questionnaire; 6 MWT: 6 min walk test, *: statistically significant.

The assessments employed to evaluate frailty reversal included a diverse array of tests, such as the rise-and-walk time test, the one-leg stand test with eyes open, the CS-30, the 6 min gait test, the 30 s stand test, the 8-foot rise-and-walk test, the Barthel index, quadriceps muscle strength, the Lawton and Brody scale, the Tinetti test, the Brief Physical Performance Battery, the Physical Performance Test, functional ambulation categories, the Physiological Profile Assessment, grip strength, the Berg Balance Scale, the 10 m walk test, habitual walking speed, standing test, physical activity levels, knee extension strength, and the five-stroke standing test. Despite some limitations noted in the interventions, these studies consistently demonstrated statistically significant improvements in the assessments used to evaluate frailty reversal, as detailed in Table 3.

### 3.6. According to Their Fragile and Pre-Fragile Community and Institutionalized Status

Hospitalized older adults in a pre-frail state who underwent lower extremity strength training significantly increased their quadriceps strength by almost one kilogram compared to the control group. In addition, these individuals demonstrated notable improvements in the 6 min walk test and the sit-to-stand test [35]. Similarly, a study with frail institutionalized older adults that implemented high- and low-intensity strength training protocols found significant improvements in the Short Physical Performance Battery (SPPB) test among the high-intensity group compared to the low-intensity and control groups [36]. Improvements in frailty status, quality of life, and participation have also been reported after a 6-week multicomponent training program [44]. In the protocol using strength training with TheraBand and calisthenics for institutionalized older adults, changes in cognitive status, independence, and quality of life were observed compared to the control group, which tended to deteriorate [41], along with a reversal of frailty in six participants from a group of pre-frail institutionalized elders [45].

For community-dwelling frail older adults, a multicomponent exercise program was utilized, including grip strength assessments, the Berg Balance Scale, the timed up and go (TUG) test, and biomarker assessments. The results indicated that the intervention group scored better than the control group, with significant improvements in both scales and biomarkers [37]. A 64% improvement in frailty scores, a 3.2-point increase in the SPPB scale, a 47% improvement in muscle power, and significant gains in lower extremity strength and aerobic capacity were observed compared to the control group [38]. The implementation of moderate-intensity step exercise programs, emphasizing the benefits of exercise and multimorbidity management, resulted in reduced fatigue, improved frailty status, and increased physical capacity and mood compared to the control group [48,49].

Research on frail and pre-frail older adults living in the community shows several positive outcomes after various exercise interventions. The use of elastic bands for strength training with pre-frail individuals led to improvements in grip strength, gait speed, and overall physical performance, along with reversals in some areas of pre-frailty [39]. With the implementation of multicomponent exercises in primary care centers, a significant reduction in the risk of falls and improvements in several outcomes were observed compared to the control group [38]. A protocol applied specifically to pre-frail women found a remarkable reversal of pre-frailty and increased strength in all the groups, with improved outcomes in gait and physical function [42].

One study incorporated vibration into their multicomponent exercise protocol, finding improvements in upper extremity strength in the dominant hand compared to the control group, along with modifications in pre-frailty domains [46]. A functional resistance program using free weights at high intensity led to improvements in gait speed, grip strength, and knee and elbow strength, with significant changes in frailty status [47]. The implementation of the X-Circuit multicomponent exercise protocol resulted in significant improvements in fitness, body composition, and a reversal of pre-frailty compared to the control group [49]. Additionally, a lower extremity muscle strengthening program at 70% of one repetition maximum (1 RM) for pre-frail women in the community led to improvements in 10 m walk times, functional capacity, knee extension strength, and TUG performance, along with significant gains in muscle power and functional capacity compared to the control group [50].

A moderate-intensity physical intervention, including endurance, functional, balance, and strength exercises, was applied. The results demonstrated significant improvements in gait speed and frailty reversal compared to the control group [40]. Following the implementation of a 3-month aerobic and resistance exercise program, with the participants divided into a supervised group and a home group guided by a booklet, an increase in knee extensor and flexor strength was observed, along with significant improvements in physical performance and gait [51]. Finally, after a 16-week program focused on low- and high-speed endurance, with a control group receiving flexibility sessions once a week, the results included a reduction in the prevalence of frailty and pre-frailty, increased lower extremity strength and power, improvements in dual-task performance for the low-speed group, and improved memory, regardless of training status [52].

## 4. Discussion

The objective of this study is to identify the characteristics and outcomes of physical exercise interventions for frail and pre-frail elderly individuals. It outlines the various training modalities, the identified physical qualities, and the parameters associated with physical exercise, including intervention duration, exercise intensity, density, progression, and frequency. Additionally, it highlights the tests and measures used to assess the reversal of frailty. The findings revealed positive and statistically significant outcomes in key measures, aligning with the study’s objectives. However, many of these studies emphasize the need for further research to provide more detailed and nuanced guidance on exercise prescriptions for frail and pre-frail older adults.

Based on the findings from the studies reviewed, it is clear that strength, power, and muscular endurance training are the most commonly used interventions for older adults. However, multicomponent exercises have also shown significant effectiveness, leading to substantial improvements. Some studies suggest that exercise programs focused on muscular strength can effectively delay the onset of sarcopenia and frailty in older adults, enhancing walking speed and the ability to rise from a chair [44,48]. A strength training program has proven to be effective, as the loss of muscle mass is directly linked to conditions like sarcopenia and a decreased ability to perform daily activities [53]. However, greater muscle strength gains have been observed with multicomponent exercises compared to concurrent programs that combine aerobic and resistance training [54]. Studies that emphasize muscular strength have shown improvements in muscle volume, balance, functional capacity, and flexibility, indicating positive outcomes for individuals experiencing frailty. Strength training programs for older adults should adhere to the same principles applied in exercise dosing for younger individuals or athletes. These principles include the considerations of overload or intensity, progression, and individuality. Such programs should provide a stimulus that exceeds the usual demands of daily activities to elicit the desired adaptive response while avoiding excessive strain [55].

Cadore et al. (2013) [56] emphasized that multicomponent exercise is one of the most effective interventions for enhancing overall physical fitness in frail individuals. Improvements in functional capacity tend to be more pronounced when interventions address multiple components of physical fitness. A study by Daniels et al. (2008) [57], which investigated programs aimed at preventing disability among frail, community-dwelling individuals, found that multicomponent programs had a greater advantage over isolated lower-extremity strength training. A systematic review revealed that 70% of the analyzed studies reported a reduction in falls, 54% showed improvements in walking speed, 80% exhibited better balance, and 70% reported increased strength among the frail individuals who participated in exercise programs focusing on strength, balance, and particularly multicomponent exercises [51].

Furthermore, the Vivifrail program [58] serves as a community- and hospital-based intervention for frailty and fall prevention. It promotes physical exercise and prescribes multicomponent exercise, improving functional capacity in older adults with frailty, especially during the pandemic [59]. However, further research is needed to establish precise dosages [38]. A study by Martinez-Velilla et al. (2019) [60] demonstrated the effectiveness of a personalized multicomponent exercise intervention that included low-intensity resistance training over a short period of 5–7 consecutive days. This approach provided significant benefits compared to the standard care and may help reverse the functional decline associated with acute hospitalization in older adults. The study by Izquierdo and Cadore (2014) [61] supports the previously mentioned dosage parameters, showing beneficial effects for older adults by incorporating exercises that mimic everyday activities, such as squats, standing and sitting exercises, and stair climbing. According to our review, exercise prescriptions should include a gradual increase in the volume, intensity, and complexity of cardiovascular endurance, muscular strength, and balance exercises. For individuals with low levels of physical activity and a history of limited exercise, it is advisable to start with moderate to high-intensity sessions at least three days a week while maintaining a relatively low training volume. Special attention should be given to functional balance and strength training to improve functional capacity, encourage adherence to the program, reduce the risk of falls, and monitor changes in older adults over time [62].

The review by Treacy et al. (2022) [26] supports the use of mobility training to enhance mobility in frail older adults living in the community. Evidence of high certainty indicates that mobility training improves mobility levels compared to control groups. Furthermore, there is moderate certainty that it may enhance overall functioning in this population, although it appears to have little to no effect on the number of falls or nursing home admissions. Similarly, a study by Lim et al. (2024) [27], which focused on a pre-frail population, found that these programs not only improve strength and physical function but also significantly increase the likelihood of reversing a pre-frail state to a robust one, with an odds ratio of 2.74 compared to minimal interventions. Additionally, the frequency of exercise sessions positively influences gait speed, suggesting that more frequent sessions may provide greater clinical benefits.

These findings highlight the importance of community-based exercise programs as a viable intervention to reduce frailty and enhance the quality of life for this vulnerable population, potentially decreasing reliance on healthcare services. It is important to note that the criterion for inclusion focused exclusively on exercise-based interventions without supplementation, which resulted in variations among the studies. Moreover, socio-cognitive strategies—such as increasing awareness of exercise benefits, demonstrating and reinforcing skills, and acknowledging achievements during each session—were effective in maintaining motivation. Research also emphasizes the critical role of social support in motivating older adults to adopt a more active lifestyle, underscoring the need to incorporate social elements into exercise programs [27].

A review by Eidam et al. (2024) [28] examined the effects of exercise and combined interventions, such as supplementation and diet, on frailty. The findings indicated that physical exercise significantly reduces the incidence of frailty, although there was a high level of heterogeneity among the studies included. Additionally, six studies were identified as having a high risk of bias, primarily due to missing outcome data and small sample sizes. Despite these limitations, the review emphasizes the growing importance of interventions aimed at preventing and treating frailty. It suggests that future research should focus on the duration and consistency of these interventions to maintain their long-term preventive effects.

Fried’s criteria are the most commonly utilized measures for assessing frailty reversal in various studies. Importantly, the Fried scale is the only measure that has established a link between frailty and mortality [63]. The Edmonton Frailty Scale, which evaluates ten domains and has a maximum score of 17 (indicating a higher degree of frailty), has also been noted. This scale has demonstrated a strong predictive capacity for two-year mortality [64] and has been both validated and applied in multiple countries [65,66]. While other frailty indices exist in the literature, they were not discussed in the articles reviewed in this research. Some of these include the frailty index [67], the clinical frailty scale [68], and various indices designed to predict falls, disability, and fractures [69].

Research on Alzheimer’s disease and its associated frailty suggests that physical exercise can yield positive outcomes [70]. Moreover, exercise-based interventions, especially when combined with nutritional recommendations [71,72] and nutritionist-led supplemental sessions [73,74], have shown favorable effects. Some studies have also examined the role of supplementation as an additional intervention [75,76]. Additionally, research focusing on functional exercise adaptations for individuals over the age of 80 with functional limitations has demonstrated significant improvements in balance over six weeks [77]. Furthermore, combining physical exercise with nutrition, polypharmacy screening, and social assessments may help prevent frailty in individuals over 80 [60].

## 5. Limitations

The studies included in this review focused exclusively on the effectiveness of exercise-based interventions, but they varied significantly in the types of exercises used. This variation creates uncertainty regarding which elements should be prioritized in the interventions, resulting in inconclusive outcomes. Additionally, this review did not have a previously published protocol. A limitation of the study is that the TIDieR checklist [78] was not formally used as a quality criterion for article inclusion, although some of its elements were considered during the planning phase. Adaptations and dosage were addressed during the extraction process and are discussed in this manuscript.

The goal of the review is to map the existing literature on the use of physical exercise in people with frailty, aiming to understand or define the characteristics of the different types of exercise employed in this population. In this context, frailty was considered an inherent condition of the population rather than a quantifiable outcome of the work methodology. No primary or secondary analysis results were available, and therefore, no quantitative recording of exercise-induced changes in the articles included in the review was conducted. This limitation prevents the methodology from being classified as a systematic review.

## 6. Conclusions

Research indicates that physical exercise is an effective intervention strategy for frail and pre-frail older adults, leading to improvements in their functionality. These benefits extend beyond older adults living in the community; they are also observed among hospitalized and institutionalized individuals, positively impacting both frailty and pre-frailty conditions. Evidence shows that exercise—including strength training, cardiovascular workouts, and multicomponent exercise—significantly enhances functional capacity, gait speed, cognitive function, and independence for hospitalized and institutionalized older adults. Six-week intervention programs have demonstrated improvements in frailty assessment scores.

Similarly, physical exercise has proven effective for frail and pre-frail older adults living in the community, resulting in enhancements in physical performance, functional capacity, fatigue, and mood. Exercise is also associated with a reduced risk of falls, improved dual-task performance, and better memory. These benefits have been reported for various exercise intensities—low, moderate, and high—over periods ranging from 5 days to 6, 12, 16, and even 20 weeks. The exercise regimens often include multicomponent activities such as vibration training, free-weight exercises, functional movements, and load-bearing exercises.

Regarding exercise program parameters, not all the studies provide precise details about the regimens. However, based on the studies analyzed, an exercise program can last from 5 consecutive days to 6 months. While specific guidelines for exercise intensity may vary, a gradual increase in both intensity and duration is recommended to improve training adherence. Exercise frequency can range from two to three sessions per week, with some programs extending to five sessions per week, depending on individual characteristics and circumstances. To maximize the benefits and maintain improvements, it is advised that physical exercise be sustained over time. Researchers also suggest that longer interventions and further studies are necessary to better understand the long-term effects of exercise on this population.

## Figures and Tables

**Table 2:** Minors scores and risk of bias for included Studies; and PEDro scores for included studies.

(**a**)													
**Author**	**A Stated Aim of the Stud**	**Inclusion of Consecutive Patients**	**Prospective Collection of Data**	**Endpoint Appropriate to the Study Aim**	**Unbiased Evaluation of Endpoints**	**Follow-Up Period Appropriate to the Major Endpoint**	**Loss to Follow-up not Exceeding 5%**	**Prospective Calculation of the Sample Size**	**A Control Group**	**Contemporary Groups**	**Baseline Equivalence of Groups**	**Statistical Analyses Adapted to Study**	**Score**
Bray, N. et al. (2020) [47]													24
Buendía-Romero et al. (2020) [45]													14
Concha-Cisternas et al. (2020) [44]													14
Losa-Reyna et al. (2019) [38]													24
(**b**)													
**Author**	**Eligibility Criteria Were Specified**	**Subjects Were Randomly Allocated to Groups**	**Allocation Was Concealed**	**The Groups Were Similar at the Baseline**		**There Was Blinding of All Subjects**	**There Was Blinding of All Therapists**	**There Was Blinding of All Assessors**	**Measures of at Least One Key Outcome (85% Subjects)**	**All Subjects Received the Treatment or Control**	**The Results of Between-Group Statistical Comparisons**	**The Study Provides Both Point Measures and Measures**	**Score**
Cardalda et al. (2019) [41]													7
Chan And Yu (2022) [48]													7
Chen et al. (2019) [39]													7
Chittrakul et al. (2020) [36]													8
Coelho-Júnior and Uchida (2021) [52]													7
Costa et al. (2020) [42]													6
Dun et al. (2022) [49]													7
Kao et al. (2022) [46]													7
Lai et al. (2021) [35]													7
Lustosa et al. (2011) [50]													7
Meng et al. (2020) [51]													6
Ng et al. (2015) [40]													9
Sadjapong et al. (2020) [37]													8
Sahin et al. (2018) [43]													5

Conventions: Green: Fully meets the criterion. Yellow: Partially meets the criterion.Red: Does not meet the criterion.

## Data Availability

Due to the type of research, online data were used.

## Supplementary Materials

The following supporting information can be downloaded at: https://www.mdpi.com/article/10.3390/geriatrics9060163/s1, Table S1. Preferred Reporting Items for Systematic reviews and Meta-Analyses extension for Scoping Reviews (PRISMA-ScR) Checklist.
